# Prenatal exposure to endocrine-disrupting chemicals and childhood atopic dermatitis: epidemiological evidence

**DOI:** 10.3389/fmicb.2025.1681214

**Published:** 2025-10-29

**Authors:** Yuxin Chen, Le Zhang, Ting Yang, Limei Chen

**Affiliations:** ^1^Department of Dermatology, Affiliated Children's Hospital of Jiangnan University (Wuxi Children's Hospital), Wuxi, Jiangsu, China; ^2^Wuxi Key Laboratory of Genetic and Metabolic Diseases in Children, Department of Paediatrics, Affiliated Children's Hospital of Jiangnan University, Wuxi Children's Hospital, Wuxi, Jiangsu, China; ^3^The Affiliated Wuxi Center for Disease Control and Prevention of Nanjing Medical University, Wuxi Center for Disease Control and Prevention, Wuxi Medical Center, Nanjing Medical University, Wuxi, Jiangsu, China

**Keywords:** endocrine disrupting chemicals (EDCs), atopic dermatitis (AD), prenatal exposure, per- and polyfluoroalkyl substances (PFAS), bisphenol A (BPA), parabens, triclosan (TCS), phthalate esters (PAEs)

## Abstract

Atopic Dermatitis (AD) is a highly prevalent chronic inflammatory disease in children, and its global prevalence is continually rising. However, data from the past decade indicate that this overall trend masks a disparity: while the prevalence has plateaued in high-income countries, it has shown a significant upward trend in low- and middle-income countries. Prenatal exposure to endocrine-disrupting chemicals (EDCs) is an environmental factor of growing scientific concern. Key EDCs of interest include per- and polyfluoroalkyl substances (PFAS), phenolics such as bisphenol A (BPA), parabens, and triclosan (TCS), as well as phthalate esters (PAEs). Although epidemiological studies indicated an association between prenatal EDCs exposure and an increased risk of offspring developing AD, key challenges remain unresolved, including population heterogeneity, methodological variations in exposure assessment, and elucidation of the underlying mechanisms. The review summarized the epidemiological evidence linking prenatal EDCs exposure to childhood AD, aiming to provide a theoretical basis for the early prevention of AD. Furthermore, it highlighted the future need to integrate multi-omics technologies with prospective cohort studies to elucidate the effects of mixed EDCs exposures and identify critical intervention windows.

## 1 Introduction

Atopic dermatitis (AD) is a chronic, recurrent inflammatory skin disease often accompanied by intense pruritus. The phenotype of AD exhibits significant heterogeneity, with the distribution and morphology of skin lesions varying with age. In infancy, it typically begins on the cheeks and extends to the trunk and extensor surfaces of the limbs, presenting primarily as acute eczema. During childhood, the lesions tend to become more localized, often involving the flexural areas or periarticular regions, with a subacute presentation and less exudation compared to the infantile stage. In adolescence, the eruptions predominantly affect the antecubital and popliteal fossae, neck, and acral regions, characterized by localized latensification and thickening (Guttman-Yassky et al., 2025). Approximately 60% of AD cases are onset in infancy, and 90% manifest before the age of five, often persisting in childhood and adolescence. The severe pruritus associated with AD skin lesions disrupts sleep and impairs growth and development in affected children, diminishing the quality of life for both the patients and their families, while also posing potential risks for behavioral and psychological comorbidities. Children with AD could develop food allergies sequentially or concurrently. Subsequently, about 50% of those with moderate-to-severe AD develop allergic rhinitis, and 34% are comorbid with asthma or other atopic conditions (Guttman-Yassky et al., 2025). Thus, AD is regarded as the “first step” in the atopic march.

The AD affects 15–30% of the global population, with its prevalence continuing to rise. Notably, the incidence of AD in the pediatric population has seen a significant increase over recent decades (Ständer, 2021). Although the development of AD is influenced by multiple factors, substantial evidence suggests that environmental factors play a non-negligible role. Traditional factors (e.g., genetics) cannot fully explain this increasing incidence, suggesting that environmental exposure could be key drivers. Within this context, prenatal exposure to endocrine disrupting chemicals (EDCs) has garnered considerable scientific interest. EDCs are a class of exogenous chemicals capable of interfering with the synthesis, metabolism, or signal transduction of endogenous hormones. They could disrupt immune and metabolic homeostasis by interfering with hormone signaling pathways (Ghassabian et al., 2022). Although traditional EDCs such as polychlorinated biphenyls (PCBs) (Peng et al., 2019; Yu et al., 2020), polybrominated diphenyl ethers (PBDEs) (Kemmlein et al., 2009; Betts, 2008), and organochlorine pesticides (Kabasenche and Skinner, 2014) have been progressively phased out, the research focus in recent years has gradually shifted toward emerging EDCs. These include per- and polyfluoroalkyl substances (PFAS), phenolics [e.g., bisphenol A (BPA), parabens, and triclosan (TCS)], and phthalate esters (PAEs). Due to their widespread use in everyday consumer goods and industrial products, the pregnant women face the risk of low-dose, chronic exposure to EDCs.

However, assessing exposure to persistent (e.g., PFAS) and non-persistent (e.g., BPA, PAEs) EDCs presents distinct challenges. For non-persistent chemicals with short half-lives, their concentrations in the human body could fluctuate significantly over short periods due to rapid metabolism and excretion following exposure. This variability implies that a single biospecimen measurement (e.g., a spot urine sample) could fail to accurately represent an individual’s long-term average exposure level, leading to misclassification of exposure status (Sugeng et al., 2020; Perrier et al., 2016). Therefore, accurate assessment strategies require frequent repeated measurements (e.g., collecting multiple urine samples) to capture temporal exposure patterns. In contrast, persistent chemicals (e.g., PFAS) have very long half-lives and could steadily accumulate in human adipose tissue or blood over years. Their internal concentrations remain relatively stable, allowing a single measurement to reasonably reflect the long-term cumulative exposure burden. The primary challenge in assessing exposure to these chemicals lies in their high persistence: they remain detectable long after exposure has ceased, which complicates the identification of critical time windows during which exposure could influence the development of specific health outcomes (Lenters et al., 2019; Melough et al., 2022).

Pregnancy and early life represent key developmental windows for fetal immune system maturation. Maternal exposure to EDCs could transfer to the fetus and neonate via the placenta or breast milk, and disrupt fetal immune programming by altering the maternal-fetal microenvironment, thereby increasing the child’s risk of developing AD (Bansal et al., 2018). In recent years, a growing body of epidemiological evidence indicated that prenatal EDCs exposure could be significantly associated with the development and progression of AD in children (Spanier et al., 2012). The review was to summarize and critically appraise current evidence on the association between prenatal exposure to the emerging EDCs and AD in children. Furthermore, it could provide a scientific basis for developing effective strategies to mitigate prenatal EDCs exposure and implementing early-life interventions for childhood AD.

## 2 The relationship between prenatal endocrine disrupting chemical exposure and atopic dermatitis in children

The diversity of EDCs and their widespread environmental distribution contribute to the complexity of exposure pathways (Table 1). In recent years, numerous studies have revealed potential associations between prenatal EDCs exposure and childhood AD. The following section summarized the epidemiological evidence from recent years linking prenatal exposure to PFAS, BPA, parabens, TCS, and PAEs with AD in children.

**Table 1:** EDCs exposure levels in pregnant women across various countries and time periods.

EDCs	Study period	Sample size	Country	Sample source	Pollutant	Detection frequency	GM (ng/mL)	Median concentration (ng/mL)	Reference
PFAS	2004	244	China	Umbilical cord serum	PFOS	99.60%	ND	5.50	Wen et al. (2019)
					PFOA	66.00%	ND	1.71	
					PFNA	59.40%	ND	2.30	
					PFHxS	89.30%	ND	0.035	
	2001–2005	863	China	Umbilical cord plasma	PFOS	89.57%	2.50	3.49	Wang et al. (2011)
					PFOA	50.75%	0.68	0.65	
	2012	615	China	Plasma	PFOS	100.00%	10.77	ND	Liang et al. (2020)
					PFOA	100.00%	19.41	ND	
					PFHxS	100.00%	2.66	ND	
	2016	789	China	Umbilical cord serum	PFOS	98.78%	ND	2.66	Zhou et al. (2024)
					PFOA	99.46%	ND	4.16	
					PFHxS	90.26%	ND	0.92	
					6:2Cl-PFESA	99.46%	ND	1.91	
	2012–2015	811	China	Umbilical cord plasma	PFOS	100.00%	ND	2.48	Chen et al. (2018)
					PFOA	99.90%	ND	6.98	
					PFNA	100.00%	ND	0.64	
					PFDA	99.10%	ND	0.36	
					PFUA	99.90%	ND	0.40	
					PFDoA	90.40%	ND	0.09	
					PFHxS	100.00%	ND	0.16	
	2003–2009	2095	Japan	Plasma	PFOS	100.00%	5.01	5.02	Okada et al. (2014)
					PFOA	100.00%	2.08	2.01	
					PFNA	99.90%	1.19	1.15	
					PFHxS	81.90%	0.275	0.296	
					PFTrDA	97.60%	0.312	0.329	
	1999–2002	1,668	America	Plasma	PFOS	100.00%	25.40	25.70	Sagiv et al. (2015)
					PFOA	100.00%	5.70	5.80	
					PFHxS	99.00%	2.50	2.40	
					PFNA	99.00%	0.60	0.70	
	1996–2002	2,139	Denmark	Serum	PFOS	100.00%	28.30	ND	Bjerregaard-Olesen et al. (2017)
					PFOA	100.00%	4.30	ND	
					PFHxS	99.90%	0.45	ND	
	2007–2009	391	Norway	Serum	PFOS	100.00%	8.00	ND	Bjerregaard-Olesen et al. (2017)
					PFOA	100.00%	1.60	ND	
					PFHxS	99.00%	0.60	ND	
BPA	2019–2020	111	China	Urine	BPA BPS BPF	100.00% 46.80% 63.1%	7.33 ND ND	7.46 ND ND	Li et al. (2021)
	2021	381	China	Urine	BPA	88.00%	0.644	0.624	Pan et al. (2024)
					BPS	100.00%	0.089	0.063	
					BPF	35.00%	0.056	ND	
	2011–2014	4,577	Japan	Urine	BPA	>60%	ND	0.40	Suwannarin et al. (2024)
					BPS	<24%	ND	ND	
					BPF	<24%	ND	ND	
	2006–2012	413	South Korea	Urine	BPA (Early pregnancy)	ND	0.79	0.75	Lee et al. (2021)
					BPA (Late pregnancy)	ND	1.19	1.11	
	2017–2019	196	South Korea	Urine	BPA	92.00%	2.10	2.10	Kang et al. (2020)
					BPS	65.00%	0.10	0.05	
					BPF	81.00%	0.20	0.20	
	1999–2006	568	America	Urine	BPA	94.00%	1.80	ND	Hoepner et al. (2013)
	1998–2017	4,454	America	Urine	BPA	ND	ND	0.96	Miller et al. (2025)
					BPS	ND	ND	0.44	
					BPF	ND	ND	0.29	
	2008–2011	2000	Canada	Urine	BPA	88.00%	0.90	ND	Arbuckle et al. (2014)
	2010–2012	846	Denmark	Urine	BPA	100.00%	ND	1.29	Mehlsen et al. (2022)
Parabens	2016	162	China	Serum	MeP	88.30%	1.86	3.080	Li et al. (2020)
					EtP	68.50%	0.239	0.327	
					Bup	0.60%	0.014	0.014	
	2022	483	China	Serum	MeP	>90%	ND	0.29	Fu et al. (2024)
					EtP	>90%	ND	ND	
					PrP	>90%	ND	0.18	
					Bup	>90%	ND	ND	
					Bzp	83.00%	ND	ND	
	2007–2010	344	Japan	Urine	MeP	94.00%	ND	108.00	Shirai et al. (2013)
					EtP	81.00%	ND	7.26	
					PrP	89.00%	ND	33.30	
	2007–2014	218	America	Urine	MeP	96.00%	ND	38.90	Kim et al. (2021)
					EtP	50.00%	ND	0.70	
					PrP	78.00%	ND	8.30	
	2010–2012	200	Denmark	Urine	MeP	95.00%	ND	20.70	Tefre de Renzy-Martin et al. (2014)
					EtP	60.00%	ND	1.01	
					PrP	83.00%	ND	4.17	
	2003–2008	629	Germany	Urine	MeP	ND	ND	38.20	Thürmann et al. (2021)
					EtP	ND	ND	2.50	
					Bup	ND	ND	0.70	
	2004–2008	120	Spain	Urine	MeP	100.00%	ND	324.00	Casas et al. (2011)
TCS	2016	162	China	Serum	TCS	96.30%	1.00	1.08	Li et al. (2020)
	2022	483	China	Serum	TCS	>90%	ND	0.19	Fu et al. (2024)
	2004–2008	120	Spain	Urine	TCS	59.50%	ND	6.10	Casas et al. (2011)
	2008–2011	2000	Canada	Urine	TCS	ND	ND	8.80	Etzel et al. (2018)
	1999–2000	601	America	Urine	TCS	70.00%	17.50	16.50	Berger et al. (2018)
PAEs	2012–2019	497	China	Urine	MBzP	90.10%	ND	0.22	Wang et al. (2025)
					MECPP	99.40%	ND	9.43	
					MMP	100.00%	ND	10.17	
					MBP	100.00%	ND	104.46	
					MEHP	100.00%	ND	1.93	
					MEHHP	100.00%	ND	8.55	
					MEP	100.00%	ND	8.04	
					MEOHP	100.00%	ND	7.66	
	2005–2008	149	Japan	Urine	MEP	100.00%	7.42	6.01	Suzuki et al. (2010)
					MEOHP	100.00%	8.60	9.20	
					MBzP	99.00%	4.27	3.46	
					MnBP	100.00%	46.20	48.10	
					MEHHP	100.00%	8.080	8.61	
					MEHP	99.00%	4.14	4.44	
					MMP	100.00%	6.95	6.49	
	2006–2012	413	South Korea	Urine	MEHHP (Early pregnancy)	ND	10.51	10.73	Lee et al. (2021)
					MEHHP (Late pregnancy)	ND	13.00	13.71	
					MEOHP (Early pregnancy)	ND	10.15	10.31	
					MEOHP (Late pregnancy)	ND	13.00	13.71	
					MnBP (Early pregnancy)	ND	32.39	36.47	
					MnBP (Late pregnancy)	ND	33.39	36.50	
	2017–2020	171	America	Urine	MEP	100.00%	24.00	ND	Buckley et al. (2022)
					MEHHP	99.00%	3.80	ND	
					MEOHP	97.00%	3.20	ND	
					MECPP	99.00%	5.00	ND	
					MEHP	96.00%	1.60	ND	
					MMP	61.00%	0.38	ND	
	2008–2011	2000	Canada	Urine	MEP	99.00%	32.02	ND	Arbuckle et al. (2014)
					MEHHP	95.00%	10.81	ND	
					MEOHP	95.00%	7.54	ND	
					MnBP	99.00%	11.59	ND	
					MEHP	95.00%	2.63	ND	
					MMP	<15%	ND	ND	
					MCHP	<15%	ND	ND	
					MiNP	<15%	ND	ND	
					MOP	<15%	ND	ND	

EDCs, endocrine disrupting chemicals; PFAS, per- and polyfluoroalkyl substances; PFOA, perfluorooctanoic acid; PFOS, perfluorooctanesulfonic acid; PFHxS, perfluorohexanesulfonic acid; PFESA, perfluoroalkyl ether sulfonic acids; PFUnDA, perfluoroundecanoic acid; PFDoDA, perfluorododecanoic acid; PFTrDA, perfluorotridecanoic acid; PFDA, perfluorodecanoic acid; PFDoA, Perfluorododecanoic acid; BPA, bisphenol A; BPF, bisphenol F; BPS, bisphenol S; MeP, methylparaben; EtP, ethylparaben; PrP, propylparaben; BuP, butylparaben; BzP, benzylparaben; TCS, triclosan; PAEs, phthalate esters; MBzP, mono-benzyl phthalate; MECPP, mono-(2-ethyl-5-carboxypentyl) phthalate; MBP, mono-butyl phthalate; MMP, mono-methyl phthalate; MEHHP, mono-(2-ethyl-5-hydroxyhexyl) phthalate; MEOHP, mono-(2-ethyl-5-oxohexyl) phthalate; MnBP, mono-n-butyl phthalate; MiNP, mono-isononyl phthalate; MnOP, mono-n-octyl phthalate; MEHP, mono-(2-ethylhexyl) phthalate; MEP, monoethyl phthalate; MCHP, mono-cyclo-hexyl phthalate; MOP, Mono-n-octyl Phthalate; GM, geometric mean; ND, not detectable.

### 2.1 Per- and polyfluoroalkyl substances (PFAS)

#### 2.1.1 Prenatal exposure to PFAS

The PFAS are a group of hydrocarbons in which hydrogen atoms are completely or partially replaced by fluorine atoms (Spyrakis and Dragani, 2023). They exhibit extreme hydrophobicity, chemical stability, and thermal stability, making them highly persistent in the natural environment (Buck et al., 2021). As a result, PFAS are widely distributed in nature, and the population—particularly pregnant women—is easily exposed to these compounds, facing potential health risks (Bangma et al., 2022). PFAS could enter the maternal body through various routes such as the skin, digestive tract, and respiratory system (Schwanz et al., 2016; Sunderland et al., 2019; Guardian et al., 2020; Franko et al., 2012). They could cross the placental barrier and accumulate in the fetus, potentially leading to adverse health outcomes such as allergic or atopic diseases (Liu et al., 2024). Traditional PFAS, including perfluorooctanoic acid (PFOA), perfluorooctanesulfonic acid (PFOS), and perfluorohexanesulfonic acid (PFHxS), have been listed under the Stockholm Convention on Persistent Organic Pollutants due to their persistence in the environment and bioaccumulative toxicity, and are being progressively phased out or restricted (Liang et al., 2022). In response, new substitutes for PFOS, such as perfluoroalkyl ether sulfonic acids (PFESA), continue to emerge (Liu et al., 2021; Xu et al., 2021). The potential toxicity of these emerging PFAS compounds, particularly their long-term effects on the health of pregnant women and fetuses, requires further in-depth research.

**Figure 1:**
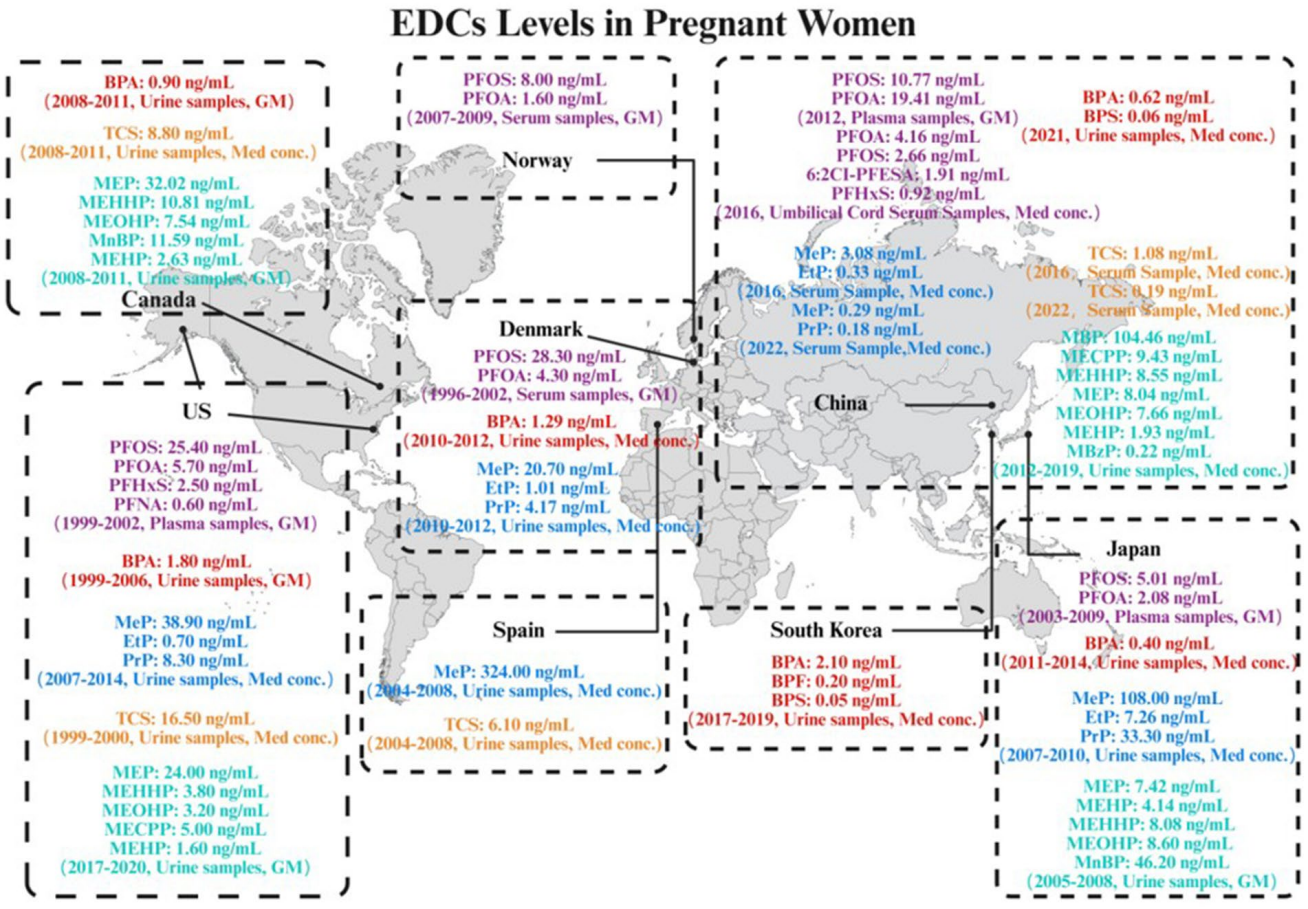
Global levels of endocrine-disrupting chemicals (EDCs) in pregnant women.

As shown in Figure 1 and Table 1, accumulating evidence indicated ubiquitous exposure to PFAS in pregnant populations. In Asian, PFOA and PFOS were detected in all plasma samples from pregnant women in China and Japan. However, compared to Japan, Chinese pregnant women demonstrated higher plasma exposure levels of PFOA and PFOS, with geometric means (GM) of 19.41 ng/mL and 10.77 ng/mL, respectively (Liang et al., 2020; Okada et al., 2014). PFOA demonstrated the highest median concentration (4.16 ng/mL) and was detectable in over 90% of umbilical cord serum samples, followed by PFOS (2.66 ng/mL) (Zhou et al., 2024). The exposure levels of PFOS and PFOA in umbilical cord plasma were found to be comparable to those in serum (Chen et al., 2018). In addition, the novel alternative 6:2Cl-PFESA was also detected at a relatively high concentration, with a median value intermediate between those of PFOS and PFHxS (Zhou et al., 2024). In a cohort study conducted in North America, PFOS and PFOA were detected in all maternal plasma samples collected from pregnant women, with PFOS exhibiting the highest concentration among all PFAS compounds, having a GM of 25.4 ng/mL (Sagiv et al., 2015). PFOS and PFOA were detected in all serum samples collected from pregnant women in Denmark and Norway. The GM for PFOS concentrations were 28.3 ng/mL and 8.0 ng/mL in Danish and Norwegian pregnant women, respectively, while the GM for PFOA were 4.3 ng/mL and 1.6 ng/mL, respectively (Bjerregaard-Olesen et al., 2017). In Europe, a clear trend of declining exposure to legacy PFAS is evident following their phase-out. Specifically, levels measured in Norwegian pregnant women (2007–2009) were significantly lower than those previously documented in Danish pregnant women (1996–2002). Collectively, global maternal exposure is characterized by dominant long-chain PFAS (PFOA/PFOS) co-occurring with short-chain analogs (e.g., PFHxS) and emerging alternatives (e.g., 6:2Cl-PFESA). These compounds could transgress the placental barrier and bioaccumulation in fetal circulation (Liu et al., 2024; Souza et al., 2020). Given their potential to disrupt offspring immune development—particularly via transplacental transfer-mediated developmental immunotoxicity—investigating associations between prenatal PFAS exposure and childhood AD has emerged as a critical research priority, warranting subsequent synthesis of epidemiological evidence and mechanistic insights.

#### 2.1.2 Prenatal PFAS exposure and childhood atopic dermatitis

Epidemiological studies on prenatal PFAS exposure and childhood AD have primarily emerged from Asian regions yet reveal notably divergent findings.

In the Taiwan, China Birth Cohort, prenatal PFOA and PFOS exposure showed no significant association with the risk of childhood AD (Wang et al., 2011). A recent meta-analysis concluded that prenatal exposure to PFOA, PFOS, perfluoroundecanoic acid (PFUnDA), perfluorododecanoic acid (PFDoDA), and perfluorotridecanoic acid (PFTrDA) showed no significant effect on childhood AD (Hatem et al., 2025).

**Figure 2:**
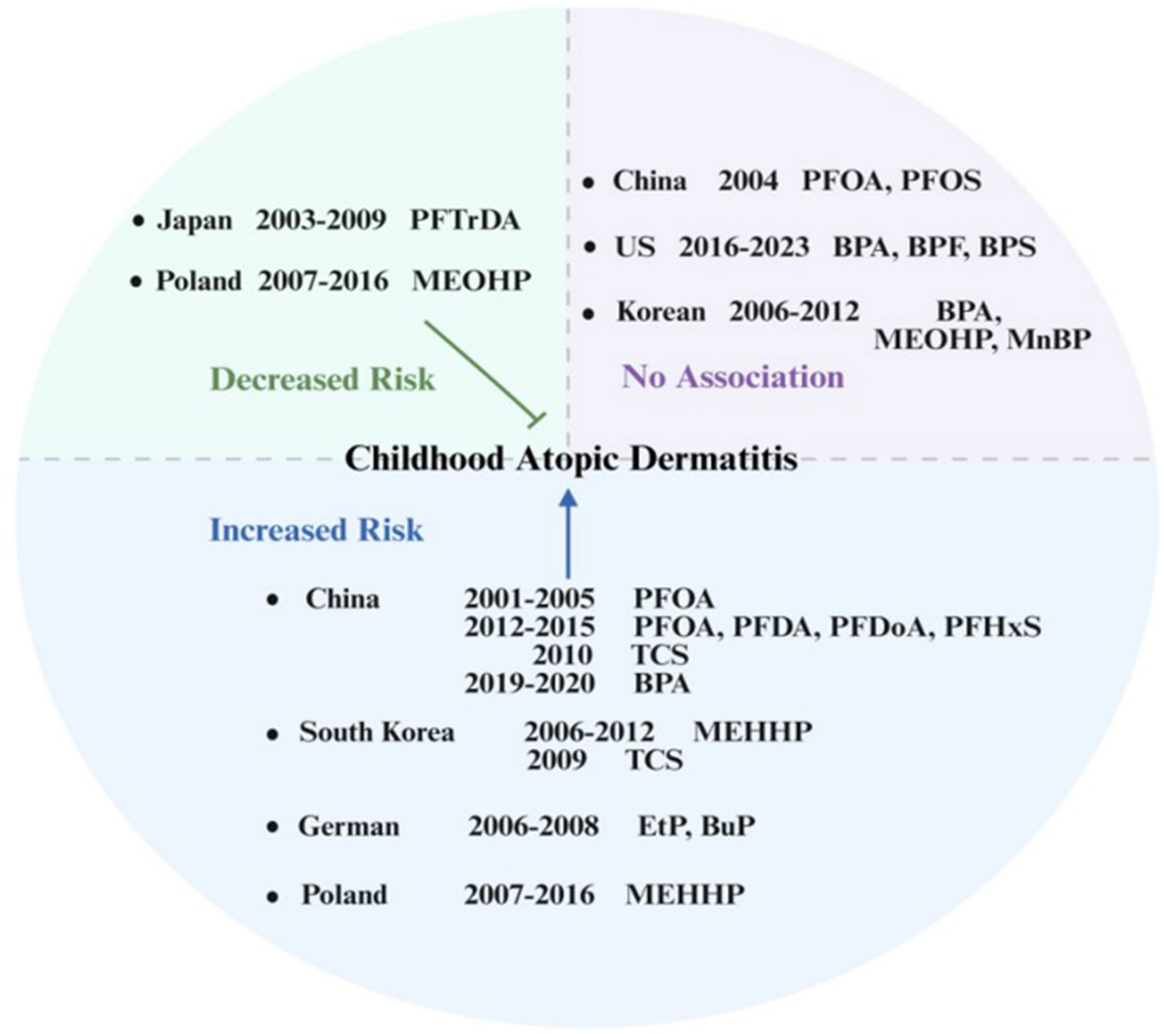
Prenatal exposure to endocrine-disrupting chemicals (EDCs) and risk of atopic dermatitis in offspring: an international perspective.

Conversely, the Hokkaido Study on Environment and Children’s Health suggested that lower prenatal exposure to PFTrDA could reduce the odds of early childhood eczema, but this association was restricted to female infants (Okada et al., 2014). Another cohort study in Taiwan, China indicated that higher prenatal PFOA exposure levels were associated with an increased odds of early-onset AD (Wen et al., 2019). Further analysis within this cohort revealed that prenatal PFOA exposure elevated the risk of AD specifically in children with glutathione S-transferase theta 1 (GSTT1) and glutathione S-transferase mu 1 (GSTM1) null genotypes (Wen et al., 2019). A prospective birth cohort study conducted in Shanghai, China demonstrated that prenatal exposure to PFOA, perfluorodecanoic acid (PFDA), Perfluorododecanoic acid (PFDoA), and PFHxS was significantly associated with an increased odds of AD in female children (Chen et al., 2018) (Figure 2).

The heterogeneity in these findings could be partly attributable to the complex nature of real-world chemical exposures, which typically occur as mixtures rather than as isolated compounds (Ribeiro et al., 2017). Health risk assessments based on single substances could overlook potential interactions between co-occurring environmental chemicals. These interactions could be dose-additive, where the combined effect equals the sum of individual effects; synergistic, where the combined effect is greater than the sum; or antagonistic, where one chemical reduces the effect of another (Couleau et al., 2015). For instance, coexposure to certain PFAS could exhibit synergistic immunotoxicity even when individual compound concentrations are below effect thresholds (Du et al., 2025). Therefore, mixture-based exposure assessment approaches are critically needed to better understand the complex etiology of childhood AD (Charles et al., 2002).

Mechanistically, prenatal PFAS exposure could enable these compounds to cross the placental barrier, leading to fetal accumulation (Liu et al., 2024; Souza et al., 2020). *In vitro* assays combined with RNA-seq analysis have revealed that PFAS could contribute to AD-like skin pathology by impairing vitamin D receptor signaling and antimicrobial defense (Kim et al., 2025). By *in vitro* assays and RNA-seq analysis PFAS could contribute to AD-like skin pathology by impairing vitamin D receptor signaling and antimicrobial defense.

Collectively, studies indicated that the association between prenatal PFAS exposure and AD exhibited complexity. Certain research suggested specific PFAS compounds (notably PFOA) could increase the risk of childhood AD, with evidence pointing toward gender- or gene-specific susceptibility. However, other studies have failed to establish significant associations. These discrepancies could stem from spatial and temporal heterogeneity in PFAS exposure levels, variations in mixture composition, and modulatory effects of host factors such as sex specificity and genetic background, necessitating further investigation into the integrated effects of chemical mixtures and their biological mechanisms to establish causal relationships.

### 2.2 Bisphenol A (BPA)

#### 2.2.1 Prenatal exposure to BPA

Bisphenol A, chemically designated as 2,2-bis(4-hydroxyphenyl)propane, is a compound featuring two phenolic hydroxyl groups and ranks among the most widely used chemicals globally (Eladak et al., 2015). It is commonly found in thermal receipt paper, dental sealants, and the linings of numerous food and beverage containers (Thoene et al., 2018). Regarding its biological properties, BPA exposure has been associated with cardiovascular disease, respiratory issues, metabolic disorders, kidney disease, and reproductive health concerns. These health risks have prompted restrictions or bans on its use in certain food or beverage packaging in some countries (Eladak et al., 2015; Lang et al., 2008). Due to these safety concerns, BPA is frequently replaced by structurally similar analogs, bisphenol F (BPF) and bisphenol S (BPS) (Eladak et al., 2015). BPF and BPS are also present in food packaging, beverage containers, and paper products. Additionally, BPF is used in pipe and tank linings to enhance durability and thickness, while BPS finds application in detergents and corrosion inhibitors (Rochester and Bolden, 2015). Significant increases in the use of these alternatives over the past decade have led to higher environmental exposure levels (Eladak et al., 2015; Rochester and Bolden, 2015). The primary route of human exposure to BPA is the ingestion of contaminated food or beverages. Dermal contact (e.g., handling thermal paper) and inhalation of BPA-containing indoor dust represent additional potential exposure pathways (Eladak et al., 2015). Given the widespread human exposure to BPA, its impact as a common EDC on vulnerable populations, particularly pregnant women and their fetuses, is a subject of significant research focus. Prenatal exposure to BPA could pose potential health risks to the developing fetus, making it a critical topic in environmental health research.

As shown in Figure 1 and Table 1, in Asian, BPA and BPS were detected in over 80% of the urine samples from pregnant women in China. BPA exhibited the highest median concentration of 0.624 ng/mL (Pan et al., 2024). In Korean pregnant women, the detection rates of BPA, BPS, and BPF in urine ranged from 65 to 92%. BPA exhibited the highest median concentration at 2.1 ng/mL (Kang et al., 2020). Among Asian countries, pregnant women in Japan exhibit relatively lower exposure levels to BPA compared to those in China and South Korea. BPA was detected in over 60% of the maternal urine samples, with a median concentration of 0.4 ng/mL, while the detection rates of both BPF and BPS were below 24% (Suwannarin et al., 2024). In North America, pregnant women in the United States exhibited higher BPA exposure levels compared to those in Canada. BPA was detectable in 94% of urine samples from U. S. pregnant women, with a GM concentration of 1.8 ng/mL. In contrast, BPA was detected in 88% of Canadian women, at a lower GM concentration of 0.9 ng/mL (Hoepner et al., 2013; Arbuckle et al., 2014). In Europe, BPA was detected in all urine samples collected from pregnant women in Denmark, with a median concentration of 1.29 ng/mL (Mehlsen et al., 2022). Collectively, these large-scale birth cohort studies from diverse global regions consistently demonstrated the high prevalence and ubiquity of BPA exposure among pregnant women. Studies spanning China (Pan et al., 2024), Korea (Kang et al., 2020), Japan (Suwannarin et al., 2024), the United States (Hoepner et al., 2013), Canada (Arbuckle et al., 2014), and Denmark (Mehlsen et al., 2022) revealed very high detection frequencies for urinary BPA, ranging from 59% to over 90%, indicating that most pregnant women were exposed to environmental BPA. Although reported median urinary concentrations exhibited some variability across studies – potentially attributable to geographic differences, population lifestyles, timing of sampling, or analytical methodologies – the widespread presence of BPA in pregnant women and its predominance among bisphenols are unequivocal and consistent findings. Notably, the South China study detected BPA and emerging bisphenols even amidst increasing use of substitutes, suggesting concurrent prenatal exposure to mixtures of BPA and structurally related phenolic compounds (Pan et al., 2024). The cumulative evidence highlighted prenatal exposure to BPA and structural analogs as a pervasive public health burden worldwide. The potential health consequences, specifically the developmental impacts on offspring, demand concerted scientific and regulatory focus.

#### 2.2.2 Prenatal BPA exposure and childhood atopic dermatitis

In this context, investigating the association between prenatal exposure to bisphenol compounds—particularly BPA—and immune-related disorders in children, such as AD, has become a critical research priority. There is limited research on the association between prenatal exposure to bisphenols and AD in children. A prospective cohort study in South Korea showed that BPA was not strongly associated with AD in the single-pollutant model (Lee et al., 2021). A United States cohort study found no association between prenatal exposure to BPA, BPF, and BPS and the risk of childhood AD. Furthermore, in girls, a higher continuous BPS exposure index during pregnancy was inversely associated with AD risk (Miller et al., 2025). A Chinese cohort study revealed that higher prenatal BPA exposure was associated with an increased risk of infantile eczema, suggesting that this could be related to the downregulation of FOXP3 gene expression in cord blood (Li et al., 2021) (Figure 2). Collectively, the association between maternal BPA exposure during pregnancy and the development of AD in children varies across countries. These discrepancies could be attributed to variations in exposure profiles, specific bisphenol analogs or population heterogeneity.

Although epidemiological studies on this topic were still limited, emerging evidence from research on BPA and allergies suggested potential mechanisms through which BPA could contribute to the development of childhood AD. These included the promotion of pro-inflammatory cytokines, increased serum IgE production, eosinophilia, a shift in the Th1/Th2 balance, and alterations in Th17 cell abundance (Weteska et al., 2023).

### 2.3 Parabens

#### 2.3.1 Prenatal exposure to parabens

Parabens, such as methylparaben (MeP), ethylparaben (EtP), propylparaben (PrP), butylparaben (BuP), and benzylparaben (BzP), are a class of compounds widely used as preservatives in cosmetics, food, and pharmaceutical products (Nowak et al., 2018; Błędzka et al., 2014). The primary routes of human exposure include ingestion of paraben-containing food/pharmaceuticals and dermal absorption from cosmetics. Additionally, their presence in dust and indoor air constitutes a potential secondary exposure source (Błędzka et al., 2014). While human exposure data and health effects remained relatively limited, animal studies demonstrated that parabens exhibited estrogenic properties (Boberg et al., 2010) and could accumulate in amniotic fluid (Frederiksen et al., 2008). Given their extensive use, potential endocrine-disrupting properties, and observed accumulation in amniotic fluid in animal models, assessing actual paraben exposure levels in vulnerable populations—particularly pregnant women—is critically important. Multiple biomonitoring studies across diverse countries and populations provided key evidence, revealing ubiquitous exposure to these compounds among pregnant women.

Biomonitoring studies from multiple countries, including China, Denmark, the United States, Japan, and Spain, consistently confirmed widespread exposure to parabens among pregnant populations (Table 1). In Asian, a Chinese cohort study detected MeP, EtP, PrP, and BuP in over 90% of serum samples, while BzP was detected in 83% of samples. Median levels of MeP (0.29 ng/mL) and PrP (0.18 ng/mL) were higher than those of EtP, BuP, and BzP (Fu et al., 2024). Analysis of maternal serum samples collected from 13 provinces in China in 2016 found the total median concentration of parabens and their metabolites ranged from 0.014 to 3.08 ng/mL. MeP and EtP were the predominant compounds, with median concentration of 3.08 ng/mL and 0.327 ng/mL, respectively. Concentrations of MeP and EtP in maternal serum from Northeastern China were significantly higher than those in other regions (Li et al., 2020). A Japanese cohort study reported that total MeP, EtP, and PrP were detected in over 80% of the participants, while total BuP was detected in 50% of the subjects. The median concentrations of MeP and PrP were 108 ng/mL and 33.3 ng/mL, respectively, whereas those of EtP and BuP were both below 8 ng/mL (Shirai et al., 2013). In North America, a cohort study conducted in California reported that MeP was detectable in 96% of maternal urine samples, while EtP was detectable in 50% of the samples. The median concentration of MeP was substantially higher (approximately 55-fold) than that of EtP. Additionally, PrP was detectable in 78% of the urine samples, with the median MeP concentration being about 4 times higher than that of PrP (Kim et al., 2021). In Europe, The Spanish birth cohort study found that MeP was detected in all maternal urine samples and exhibited the highest median concentration among the parabens analyzed (Casas et al., 2011). A Danish child cohort study revealed median maternal urinary concentrations of MeP and EtP as 20.7 ng/mL and 1.01 ng/mL, respectively (Tefre de Renzy-Martin et al., 2014). MeP was the most frequently detected compound and generally exhibited the highest concentrations. These studies demonstrated extremely high detection frequencies for MeP in maternal serum or urine, and its median or GM concentration was often significantly higher than other parabens (e.g., EtP, PrP, BuP, and BzP). PrP also showed high detection frequencies and concentration levels in maternal serum or urine (Fu et al., 2024; Li et al., 2020; Shirai et al., 2013; Kim et al., 2021; Casas et al., 2011; Tefre de Renzy-Martin et al., 2014). Notably, exposure levels exhibited geographical variation, exemplified by significantly higher serum concentrations of MeP and EtP in pregnant women from Northeastern China compared to other regions (Li et al., 2020). The heterogeneity in paraben exposure levels observed in pregnant women from multinational studies is primarily attributed to divergences in research methods (analytical techniques, target analytes, study period), geographical environments, and population behaviors. These findings collectively demonstrated that, despite existing regulatory measures, pregnant women remained widely exposed to parabens, with MeP and PrP representing the predominant exposure species. Given the ubiquitous presence and notable concentrations of these compounds in pregnant women, combined with their potential association with adverse health outcomes in children, such as AD, the ongoing biomonitoring of paraben exposure levels in this population is of critical importance.

#### 2.3.2 Parabens exposure and childhood atopic dermatitis

A German cohort study evaluated the association between prenatal paraben exposure and childhood AD. The study demonstrated that prenatal exposure to EtP and BuP was associated with an increased risk of very early-onset AD (diagnosed by age two) that persisted beyond this age without remission (Thürmann et al., 2021) (Figure 2).

According to the “hygiene hypothesis,” early-life microbial exposure promotes immune system development, thereby reducing the risk of allergic diseases (Ege, 2017). Conversely, direct exposure to antimicrobial compounds via oral or dermal routes may alter microbial communities in the skin or gut, potentially inducing a hypersensitive state in immune receptors within these organs and increasing the risk of hypersensitivity disorders such as asthma and eczema (Stiemsma and Turvey, 2017). It is proposed that parabens, as antimicrobial compounds, could increase the risk of AD in children prenatally exposed to them through precisely this mechanism: by traversing the placental barrier, these antimicrobial compounds have the potential to disrupt the maternal microbiota and shape the infant’s developing microbiome, thereby influencing a key factor in AD pathogenesis (Jackson-Browne et al., 2019). Beyond their potential effects on the microbiome, long-term exposure to parabens could disrupt keratinocyte differentiation, suggesting a direct interference with skin development processes (Ishiwatari et al., 2007).

Collectively, these findings indicated that gestational exposure to parabens represented a significant environmental risk factor for the development of severe and persistent early-childhood AD.

### 2.4 Triclosan (TCS)

#### 2.4.1 Prenatal exposure to TCS

Triclosan is a polychlorinated bisphenolic compound characterized by a distinct aromatic odor. As a broad-spectrum lipophilic antimicrobial agent, it is extensively used in products such as hand sanitizers, cosmetics, preservatives, and disinfectants. Consequently, TCS is frequently detected in the environment and has become an almost ubiquitous contaminant (Weatherly and Gosse, 2017; Alfhili and Lee, 2019). The primary routes of TCS exposure in pregnant women include dermal contact (e.g., using TCS-containing personal care products) and direct ingestion of contaminated drinking water, food, or animal-derived products. TCS has been frequently detected in maternal urine, blood, breast milk, and even amniotic fluid (Milanović et al., 2023; Chen et al., 2019; Yueh and Tukey, 2016), indicating that its exposure during pregnancy poses non-negligible risks to fetal health. As a widely used phenolic compound, TCS exhibits endocrine-disrupting properties (Liu et al., 2022), raising particular concern over its potential effects during critical developmental windows, especially in gestation. Assessing TCS exposure levels and associated health risks in pregnant populations is essential for understanding its developmental toxicity mechanisms. To date, multiple biomonitoring studies focusing on pregnant women have provided valuable data for such risk characterization.

Multiple pregnancy cohort studies from China, Spain, Canada, and the United States confirmed widespread TCS exposure in pregnant populations. As shown in Figure 1 and Table 1, in Asian, a 2016 Chinese cohort study detected TCS in 96.3% of maternal serum samples. The highest concentration was observed in North China (GM: 1.184 ng/mL), followed by Northeast China, Southwest China, East China, and Central-South China, in descending order of GM (Li et al., 2020). A 2022 Chinese cohort study detected TCS in over 90% of serum samples, with a median maternal level of 0.19 ng/mL (Fu et al., 2024). In Europe, Spanish birth cohort study detected TCS in only 59.5% of samples, with a median concentration of 6.1 ng/mL (Casas et al., 2011). In North America, a Canadian prospective cohort study reported a higher median concentration of TCS in maternal urine compared to that reported in a Spanish cohort (Etzel et al., 2018). A longitudinal birth cohort study in California detected TCS in 70% of maternal urine samples, with the median concentration being the highest among reported national data at the time (Berger et al., 2018). Although reported detection frequencies and concentration levels vary significantly across studies—likely reflecting differences in geography, population habits, detection methods, or sample type (serum/urine)—the detectability of TCS in maternal biological samples is a clear and prevalent phenomenon. Notably, regional variations in TCS concentrations (e.g., relatively higher levels in North China) were observed even within studies conducted in the same country. In summary, multinational evidence indicated that prenatal TCS exposure was prevalent but exhibited significant geographic heterogeneity. Given this variability in exposure levels and the potential link to childhood AD, further investigation into its determinants and health impacts—particularly regarding its contribution to offspring AD risk—constitutes a critical public health priority.

#### 2.4.2 TCS and childhood atopic dermatitis

Two Asian studies suggested that the association between TCS exposure and childhood AD could exhibit age and sex specificity. A 2009 Korean population-based cross-sectional study demonstrated a positive association between urinary TCS levels and AD in school-aged children (Choi et al., 2024). The Taiwan, China 2010 cohort study found that TCS levels were significantly associated with AD in boys (Lin et al., 2022), which indicated that the immunomodulatory effects of TCS could be regulated by developmental stage and sex factors (Figure 2).

An Animal study further elucidated the underlying mechanisms: TCS exacerbated AD-like skin inflammation in mice by inducing skin inflammation and immune cell infiltration via thymic stromal lymphopoietin (TSLP) (Schuppe et al., 2025).

In summary, exposure to TCS was robustly associated with the development of AD in children. However, more longitudinal cohort studies are warranted to confirm this causal relationship, and further investigation is needed to elucidate the underlying mechanisms.

### 2.5 Phthalate esters (PAEs)

#### 2.5.1 Prenatal exposure to PAEs

Phthalate esters are a group of compounds used as plasticizers (Zhang et al., 2021). Based on the length of their alcohol side chains, they could be categorized into long-chain PAEs—such as di(2-ethylhexyl) phthalate (DEHP), butyl benzyl phthalate (BBzP), and diisononyl phthalate (DiNP)—and short-chain phthalates, which is phthalate metabolites (mPAEs), including diethyl phthalate (DEP) and di-n-butyl phthalate (DnBP). Long-chain PAEs are predominantly used in polyvinyl chloride (PVC) products such as building materials and food containers, whereas mPAEs are more commonly found in non-PVC applications, including adhesives and personal care products. Diet is considered the primary source of exposure to long-chain PAEs during pregnancy, whereas significant sources of mPAEs, could include personal care products and indoor air (Chang et al., 2021; Mariana et al., 2016). PAEs are clearly classified as EDCs. Upon entering the body, they could disrupt the endocrine system by binding to molecular targets and interfering with hormonal homeostasis (Chang et al., 2021; Mariana et al., 2016). There is growing concern regarding the health impacts of gestational exposure to PAEs on fetal development. This growing concern was substantiated by biomonitoring data from birth cohort studies across the globe, which demonstrated widespread exposure to these toxicants.

As shown in Figure 1 and Table 1, a birth cohort study in China, revealed widespread exposure to mPAEs in pregnant women. Except for mono-benzyl phthalate (MBzP), detected in 90.1% of participants, and mono-(2-ethyl-5-carboxypentyl) phthalate (MECPP), detected in 99.4%, all other metabolites were detected in 100% of the subjects. Among the eight major mPAEs quantitatively analyzed, the concentration distribution showed a distinct hierarchy: mono-butyl phthalate (MBP) was the dominant metabolite by a large margin (median concentration: 104.46 ng/mL), with levels approximately 10 times higher than those of the second most abundant metabolite, mono-methyl phthalate (MMP). The remaining metabolites followed a descending concentration order: MECPP > mono-(2-ethyl-5-hydroxyhexyl) phthalate (MEHHP) > monoethyl phthalate (MEP) > mono-(2-ethyl-5-oxohexyl) phthalate (MEOHP) > mono-(2-ethylhexyl) phthalate (MEHP) > MBzP (Wang et al., 2025). MMP, MEP, mono-n-butyl phthalate (MnBP), MBzP, MEOHP, and MEHHP were detected in all urine samples from pregnant women in Japan. Among the monoester metabolites analyzed, urinary MnBP exhibited the highest concentration (median concentration: 48.1 ng/mL), whereas mono-isononyl phthalate (MiNP) and mono-n-octyl phthalate (MnOP) were present at considerably lower levels (Suzuki et al., 2010). In North America, a large US cohort study detected MEP, MEHHP, MECPP, MEHP and MEOHP in over 90% of the study population, with GM ranges of 1.6–24 ng/mL (Buckley et al., 2022). MEP, MEHHP, MnBP, MEHP and MEOHP were widely detected in over 90% among pregnant women in Canada. The highest measured concentrations of mPAEs were MEP (GM: 32.02 ng/mL) and MnBP (GM: 11.59 ng/mL) (Arbuckle et al., 2014). Synthesizing cohort studies from China, the United States, Canada, and Japan revealed that mPAEs exposure was globally prevalent in pregnant populations, but systematic differences existed in the dominant metabolite species and concentration levels across regions. Collectively, these findings demonstrated complex geographic heterogeneity in prenatal PAEs exposure across global populations, with short-chain metabolites such as MnBP dominating the exposure profile in most cohorts. Given that high-abundance metabolites (e.g., MnBP, MEHHP) have been shown to disrupt Th1/Th2 immune homeostasis and elevate AD risk (Lee et al., 2021; Berger et al., 2018), the nexus between their ubiquitous exposure patterns and suspected health implications necessitates further mechanistic investigation.

#### 2.5.2 PAEs and childhood atopic dermatitis

Regarding the association between prenatal PAEs exposure and childhood AD, studies suggested that metabolite type, exposure timing (window), and mixture effects collectively determine the direction of risk. A Polish prospective mother–child cohort study indicated that higher prenatal urinary concentrations of MEOHP were associated with a reduced risk of AD, whereas higher concentrations of MEHHP were associated with an increased AD risk (Podlecka et al., 2020). A Korean prospective birth cohort study, measuring maternal urinary MEHHP, MEOHP, and MnBP concentrations in early and late pregnancy, showed that exposure to MEHHP in late pregnancy was significantly associated with AD incidence. Furthermore, co-exposure to BPA and mPAEs during pregnancy synergistically increased AD risk (Lee et al., 2021) (Figure 2).

Mechanistically, metabolites such as MEHHP acted as co-stimulators that promote IL-4 and IFN-*γ* secretion, reduced the Th1/Th2 ratio *in vitro* (Pei et al., 2014), and enhanced allergen-specific IgE production (Han et al., 2014). Animal models confirmed that DEHP activated PPARα, driving Th1/Th2 balance toward Th2 polarization (Larsen and Nielsen, 2007). This mechanistic evidence elucidated a plausible pathway linking PAEs exposure to childhood AD onset.

In summary, PAEs exhibited bidirectional immunomodulatory effects on AD pathogenesis. Their net impact depends on the exposure profile composition, critical developmental windows, and immune microenvironment crosstalk, necessitating future isomer-resolved analyses, which distinguish between individual molecular structures, and mixture-based risk assessments to reconcile conflicting evidence.

## 3 Prevention and intervention measures

Given that both epidemiological and mechanistic studies suggested an association between prenatal EDCs exposure and childhood AD risk, implementing a multi-level, perinatal integrated intervention strategy is crucial (Wen et al., 2019; Lee et al., 2021; Miller et al., 2025; Kim et al., 2024). Primary measures focus on source avoidance to reduce maternal EDCs exposure levels. Pregnant women should actively modify lifestyles, such as avoiding plastic food containers containing BPA (e.g., packaging, bottles) in favor of safer materials like glass or stainless steel, and choosing personal care and household products (e.g., skincare, detergents) free of PAEs to minimize dermal absorption risk (Vandenberg et al., 2012). Dietarily, prioritizing organic produce helps reduce pesticide residue exposure, while minimizing processed food intake avoids contact with preservatives like parabens (Berni Canani et al., 2024; Çakmakçı and Çakmakçı, 2023). Secondly, nutritional interventions during pregnancy are vital for enhancing maternal-fetal barrier function and immune regulation. Supplementation with omega-3 polyunsaturated fatty acids may exert protective effects by suppressing maternal inflammatory responses and modulating fetal immune development (Gutiérrez et al., 2019). Vitamin D supplementation could reduce offspring AD risk by regulating Treg/Th2 immune balance (Martineau et al., 2017). For pregnant women at high risk of EDCs exposure (e.g., occupational exposure or residing in industrial pollution zones), dynamic monitoring of exposure levels is recommended. Regular testing of serum or urine for EDCs metabolites (e.g., BPA, mPAEs), combined with assessment of clinical biomarkers (e.g., cord blood IgE, IL-4) for early risk stratification, aids in identifying high-risk individuals for targeted interventions. At the medical intervention level, supplementation with specific probiotic strains (e.g., Lactobacillus, Bifidobacterium) shows promise. They could inhibit fetal Th2-type immune skewing by modulating the maternal gut microbiota-immune axis, although evidence-based selection of effective strains is essential (Azad et al., 2013). Simultaneously, household environmental controls, such as reducing indoor dust mites and avoiding tobacco smoke, effectively mitigate ongoing childhood EDCs exposure and allergen trigger risks. Finally, robust policy and regulation form the cornerstone of population health protection. It is critical that such policies evolve to address exposure to chemical mixtures, as humans are consistently exposed to complex combinations of EDCs (e.g., PAEs and PFAS) that could act additively or synergistically to increase AD risk. Regulatory frameworks should therefore incorporate mixture-based risk assessment approaches, establishing exposure limits based on cumulative risk and regulating classes of chemicals—such as PAEs or bisphenols—as groups rather than individual substances. Promoting comprehensive EDC regulatory legislation, such as emulating and expanding upon the EU REACH (Registration, Evaluation, Authorization and Restriction of Chemicals) framework to restrict mixture exposures in toys and mother-infant products, is essential. Strengthening public health education also holds profound significance for enhancing maternal awareness of EDCs and reducing overall population exposure levels.

## 4 Conclusion and future perspectives

Current research robustly demonstrated that prenatal exposure to multiple EDCs could significantly increase the risk of AD in children. Although epidemiological studies exhibited heterogeneity, they provided evidence for dose-dependent associations between prenatal exposure to specific EDCs and the risk of childhood AD. To effectively address this challenge, preventive strategies must integrate multi-level approaches: ranging from individual-level measures such as exposure avoidance and nutritional optimization to clinical biological monitoring and risk stratification, and further to societal-level policy support and public education. Together, these efforts should establish a comprehensive protection system covering the entire ‘pregnancy to postpartum’ continuum.

Looking ahead, research should focus on several key directions: First, in-depth investigation into the complex synergistic or antagonistic effects of mixed EDCs exposures and their impact on childhood AD, which better reflects real-world exposure scenarios. Second, elucidating the underlying mechanisms through which prenatal EDCs exposure contributes to the pathogenesis of AD in children, utilizing large international birth cohorts (e.g., COPSAC, CHAMACOS) for integrated multi-omics analysis (Maitre et al., 2022). This approach is central to uncovering the mechanisms linking early-life exposure to disease. By conducting multi-omics analyses on biological samples collected from these cohorts and correlating the findings with experimental studies, we could ultimately clarify how prenatal EDC exposure drives the development of AD by regulating gene pathways related to immune programming and skin barrier function. Third, actively exploring the feasibility of novel targeted intervention technologies based on the mechanistic research, such as the development of specific nano-detoxifying agents or receptor antagonists. By fostering deep collaboration across various disciplines—including environmental medicine, immunology, epigenetics, toxicology, and public health—and establishing a comprehensive prevention and control network based on an integrated “exposome-immune programming-clinical phenotype” model, we could potentially develop more precise and effective solutions to reduce the global disease burden of childhood AD.
